# Safety and Efficacy of Keratorefractive Lenticule Extraction (KLEx) for High Myopia with the CLEAR Platform

**DOI:** 10.3390/jcm15176585

**Published:** 2026-08-26

**Authors:** Matteo Posarelli, Lorenzo Azzaro, Stefania V. Fields, Tara Moore, Antonio Leccisotti

**Affiliations:** 1 Siena Eye Laser, 53036 Poggibonsi, Italy; 2 Centre for Research and Education in Refractive Surgery, 53035 Siena, Italy; 3 School of Biomedical Sciences, Ulster University, Coleraine BT52 1SA, UK; tara.moore@ulster.ac.uk

**Keywords:** CLEAR, KLEx, refractive surgery, laser vision correction, high myopia, astigmatism

## Abstract

**Objectives:** To assess the safety and efficacy of Keratorefractive Lenticule Extraction (KLEx) in patients with myopia ≥ 5 diopters (D). **Methods:** Retrospective study of KLEx procedures performed at Siena Eye Laser (Poggibonsi, Italy) with the CLEAR platform of the Ziemer Z8 femtosecond laser. **Results**: We included 137 eyes of 85 patients; mean age was 33.8 ± 8.3 years. Assessment at baseline showed a mean myopic error of −7.3 ± 1.4 D, a mean astigmatic error of 1.1 ± 0.8 D, and a mean spherical equivalent (SE) of −7.8 ± 1.6 D. Preoperative corrected distance visual acuity (CDVA) was 0.0 logMAR or better in all eyes. Mean corneal thickness was 510.0 ± 26.9 µm. At post-operative day 1 (POD1) uncorrected distance visual acuity (UDVA) was 0.1 ± 0.1 logMAR, with a mean residual SEQ of 0.1 ± 0.8 D. At 6 months follow-up, mean UDVA significantly increased to 0.0 ± 0.0 logMAR (*p* < 0.001), with a mean residual SE of −0.12 ± 0.7 D (*p* > 0.05 compared to POD1). At 24 months, the mean UDVA remained stable at 0.0 ± 0.0 logMAR, with a mean residual SE of −0.1 ± 0.4 D as manifest refraction (all *p* > 0.05 compared to 6 months). At 6 months and 2 years, the observed residual stromal bed (RSB) was 30 microns thicker than the RSB calculated by the Ziemer LDV Z8 software 2022. The difference between predicted (POZ) and effective (EOZ) optical zone correlated with the degree of myopia corrected (r = 0.6, *p* < 0.001). The difference between POZ and EOZ inversely correlated with astigmatic correction (r = −0.5, *p* < 0.001). **Conclusions**: In patients with high myopia, CLEAR showed good predictability and efficacy. Both UDVA and SE improved during the first 6 months, and remained stable at 2 years. The effective RSB was thicker than expected, and patients with high astigmatism achieved a larger EOZ.

## 1. Introduction

High myopia is defined as a spherical equivalent of −5.00 D or worse, and it represents a growing public health concern worldwide [1,2,3]. Global projections estimate that by 2050 nearly 10% of the world’s population, corresponding to almost one billion individuals, will be affected by high myopia [2]. Beyond the optical burden of a significant refractive error, high myopia is associated with an increased lifetime risk of sight-threatening complications, including retinal detachment, myopic maculopathy, cataract, and glaucoma [4]. Correction of high myopia therefore represents a clinically relevant challenge, and effective surgical options are needed that can safely address large refractive errors while minimizing long-term ocular morbidity.

Recent advances in refractive surgery have led to the widespread adoption of femtosecond laser technology for the correction of myopia and astigmatism [5]. Among the latest developments, keratorefractive lenticule extraction (KLEx) has emerged as a minimally invasive alternative to femto-LASIK (laser in situ keratomileusis), providing comparable refractive outcomes while offering several theoretical and clinical advantages. First, by performing the refractive correction within the deeper corneal stroma, KLEx minimizes damage to the corneal nerves and better preserves the subbasal nerve plexus, potentially reducing postoperative dry eye symptoms [6]. Second, because the procedure does not require the creation of a corneal flap, it is thought to preserve corneal biomechanics more effectively and may lower the risk of postoperative ectasia [7]. Finally, KLEx enables the correction of higher refractive errors while preserving a greater amount of corneal stromal tissue, thereby reducing the proportion of tissue removed during surgery [8,9]. For patients with very high refractive errors who are not suitable candidates for corneal refractive surgery, for example because of insufficient residual stromal thickness or abnormal corneal tomography, phakic intraocular lens implantation remains a valuable alternative, and has shown good safety and predictability in this population [10]. However, in eyes with adequate corneal thickness and normal topography, KLEx offers the advantage of avoiding an intraocular procedure.

Among the currently available KLEx platforms, CLEAR (Corneal Lenticule Extraction for Advanced Refraction), performed with the Ziemer Z8 femtosecond laser (Ziemer Ophthalmic Systems AG, Port, Switzerland), has demonstrated favorable safety and efficacy profiles for the treatment of myopia and astigmatism using low-energy laser settings [9,11,12,13,14]. Since its clinical introduction in April 2020, however, published evidence remains limited, and the available studies have mainly investigated heterogeneous patient populations with a broad spectrum of refractive errors. Our group previously reported that, for the same optical zone diameter, CLEAR requires less stromal tissue removal than femto-LASIK, making it particularly suitable for the correction of high myopia [9,12]. Therefore, the aim of this retrospective study was to evaluate the safety and efficacy of the CLEAR procedure in patients presenting with high myopia.

## 2. Methods

This was a retrospective, longitudinal, single center study conducted at Siena Eye Laser, Centre for Research and Education in Refractive Surgery (Poggibonsi, Siena, Italy). We included 137 eyes of 85 patients with preoperative myopia ≥ −5 diopters (D) that underwent CLEAR between 2022 and 2025. Inclusion criteria were: myopia ≥ −5.00 D, age ≥ 18 years; corrected distance visual acuity (CDVA) equal or better than 0.1 logMAR (Snellen: 20/25); no history of corneal infections, intraocular inflammations, previous ocular surgeries, or autoimmune systemic diseases; normal corneal tomography. Exclusion criteria were: abnormal corneal tomography; concurrent ocular comorbidities (such as, for example, glaucoma, retinal diseases, cataract); collagen or dermal autoimmune diseases; pregnancy. This study adhered to the tenets of the Declaration of Helsinki.

At baseline and follow-up, all patients underwent CDVA measurement with manifest and cycloplegic refraction, slit lamp examination, non-contact corneal tonometry, pupil dilation and full retinal examination, corneal tomography with anterior segment optical coherence tomography (AS-OCT) MS-39 (CSO, Florence, Italy), and non-invasive tear break up time (NIBUT) with Scheimpflug camera-Placido disk device (Sirius, CSO, Florence, Italy).

### 2.1. Surgical Technique

**Figure 1:**
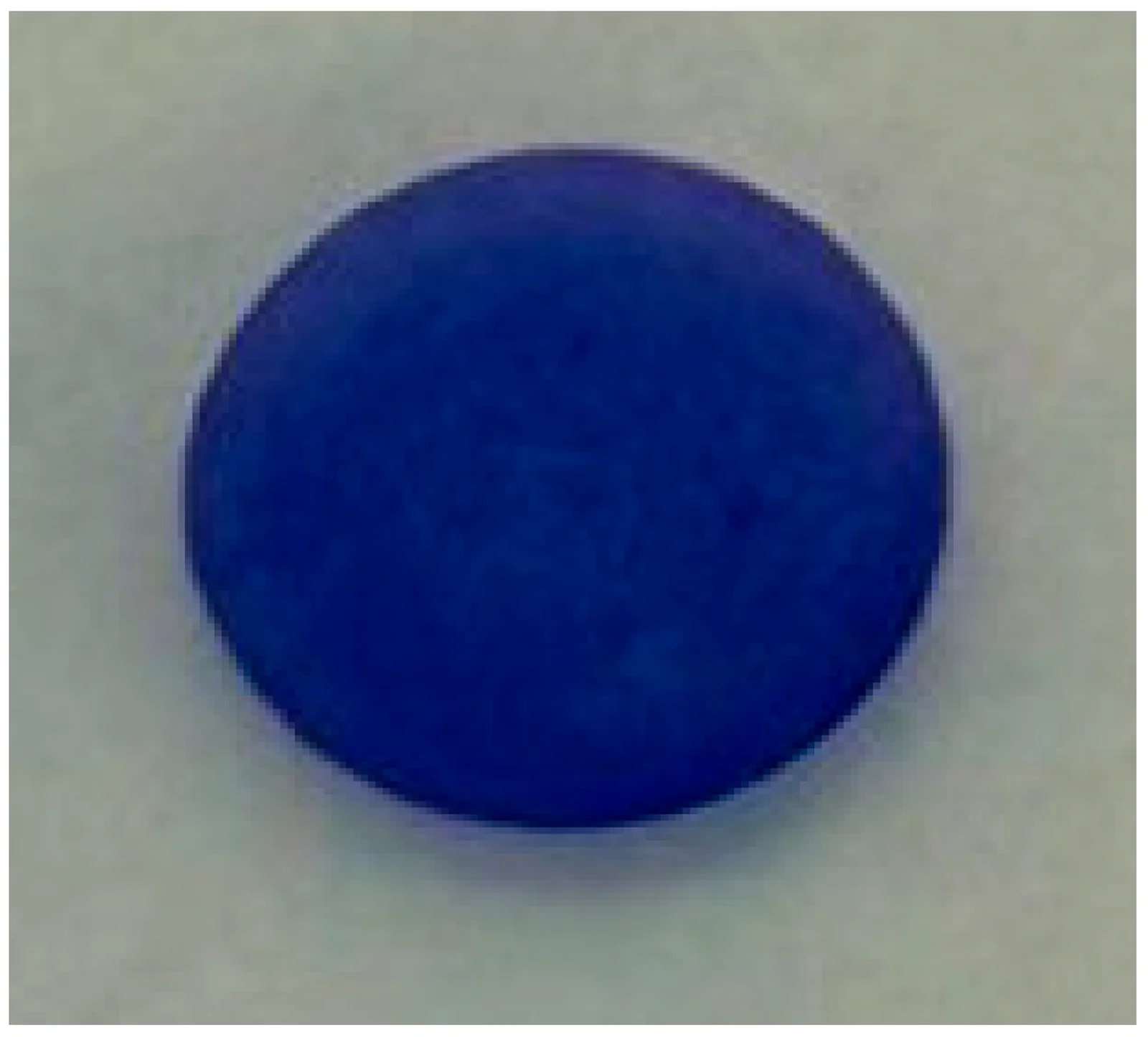
Lenticule staining using methylene blue, to visualize the lenticule margins and to confirm its integrity.

The refractive error and optical zone were set according to our nomogram, leaving a residual stromal bed (RSB) ≥ 250 µm. The Z8 laser was set to perform a single incision superotemporal (right eye) or superonasal (left eye). The diameter of the suction ring was chosen based on the white-to-white diameter measured by AS-OCT: a 9.5 mm ring was if white-to-white measurement was ≥12 mm, and a 9 mm ring was chosen for smaller diameters. Treatment was centered on the coaxially observed corneal light reflex. After completion of the lenticule cut by the femtosecond laser, the incision was opened and we used the Reinstein Lenticule Separator (Malosa MMSU1297S, BVI, Waltham, MA, USA) to dissect the anterior and posterior plane of the corneal lenticule. The lenticule was then extracted with disposable tying forceps (Malosa MMSU1414, BVI, Waltham, MA, USA) and stained with methylene blue to assess its integrity and regularity of lenticule margins (Figure 1). Postoperative topical treatment prescribed included association of 0.1% dexamethasone and 0.3% netilmicin, four times a day for 7 days and then twice a day for another 7 days, and preservative-free 0.2% sodium hyaluronate as needed. 

### 2.2. Statistical Analysis

We tested for normality on all parameters. For comparisons between baseline and follow-up we used paired *t*-test. The Pearson correlation test was used for correlation analysis. Statistically significant values were considered for *p* < 0.05. As previously described [15], the effective optical zone (EOZ) was calculated as the difference between pre-operative and post-operative tangential maps. The mean between long and short axes was considered the average EOZ.

## 3. Results

We included 137 eyes of 85 patients; 52 (61.1%) were female and 33 (38.9%) were male, with a mean age of 33.8 ± 8.3 years. At baseline, mean CDVA was 0.0 logMAR (Snellen: 20/20), with a mean myopic error of −7.3 ± 1.4 D, a mean astigmatic error of 1.1 ± 0.8 D, and a spherical equivalent (SE) of −7.8 ± 1.6 D. Corneal tomography parameters, assessed with AS-OCT, showed a mean corneal thickness of 510.0 ± 26.9 µm, a mean epithelial thickness of 55.3 ± 4.2 µm, and a mean corneal anterior curvature of 44.7 ± 1.7 D. Mean cap set thickness was 121.5 ± 8.2 µm with a mean optical zone of 6.2 ± 0.2 mm. Nine eyes were lost to follow-up by 6 months, with an additional five eyes lost between 6 months and 2 years, resulting in a cumulative loss to follow-up of 14 eyes at 2 years.

### 3.1. Visual Acuity and Efficacy

**Figure 2:**
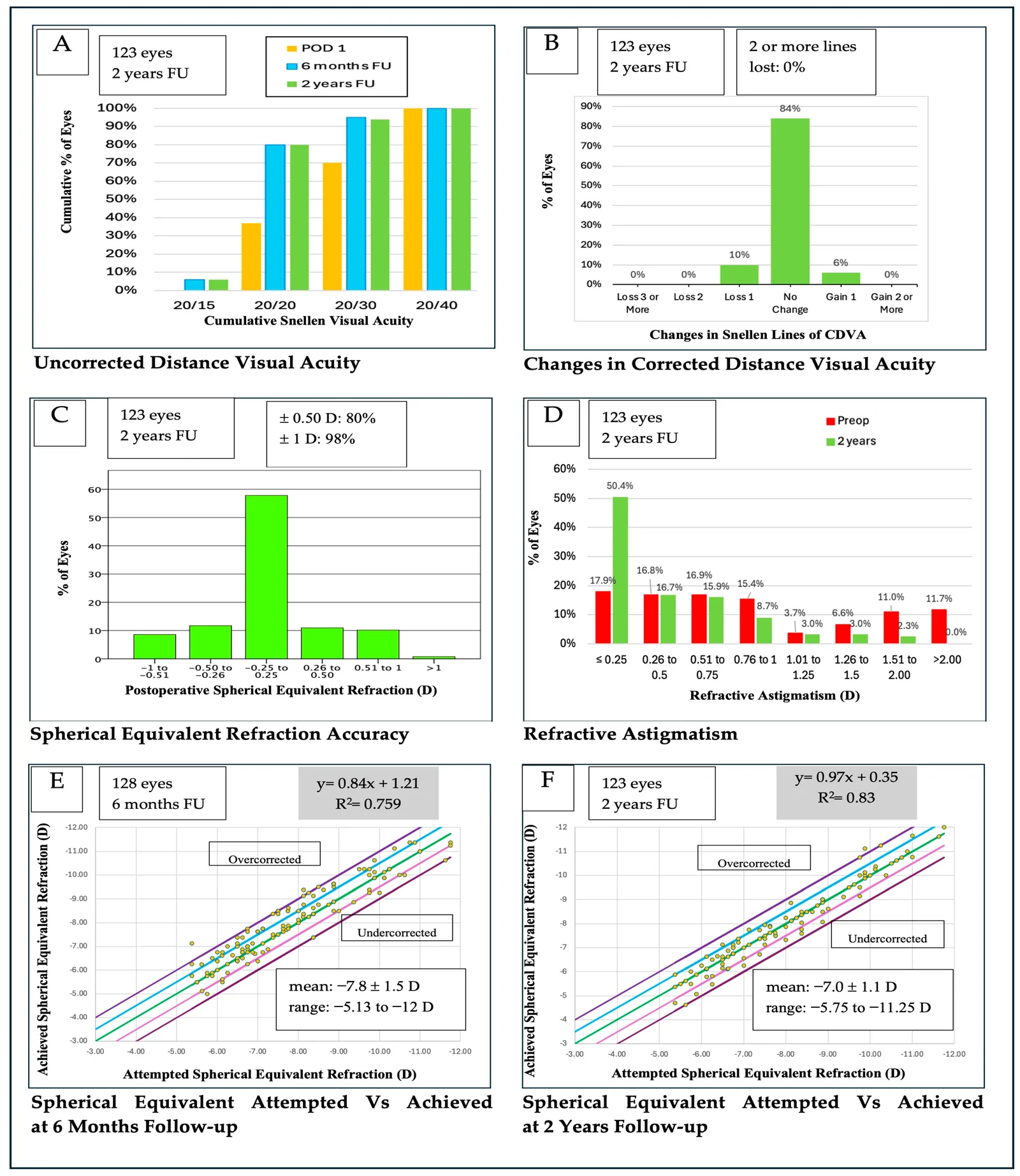
Six standard graphs reporting refractive results of lenticule extraction using Ziemer Z8 femtosecond laser. (**A**): UDVA showing stability of the visual acuity at 2 years follow-up. (**B**): changes in CDVA. (**C**): SE accuracy, showing that 80% are within ±0.50 D of residual error. (**D**): refractive astigmatism. (**E**): SE attempted versus achieved at 6 months. (**F**): SE attempted versus achieved at 2 years follow-up.

At postoperative day 1 (POD1), the mean UDVA was 0.1 ± 0.1 logMAR (Snellen: 20/25), with a mean residual spherical error of 0.15 ± 0.7 D, a mean residual cylinder error of −0.10 ± 0.8 D, and a residual SE of 0.1 ± 0.8 D. Moreover, 47.7% of the eyes had a residual refractive error between ±0.25 D, and 62.5% were between ±0.50 D. At 6 months, UDVA significantly improved to 0.0 ± 0.0 logMAR (*p* < 0.05 compared to POD1), with 94.7% of the subjects seeing 0.1 logMAR (Snellen: 20/25) or better. The mean residual sphere error was 0.0 ± 0.6 D, the residual cylinder error was −0.3 ± 0.7 D, and the mean residual SE was −0.12 ± 0.7 D (all *p* > 0.05 compared to POD1). At 24 months of follow-up, 91% of patients had a visual acuity of 0.0 logMAR or better (Snellen: 20/20), and 96% were seeing 0.1 logMAR or better (Snellen: 20/25) (Figure 2A). The mean UDVA remains stable at 0.0 ± 0.0 logMAR (Snellen: 20/20), with a mean residual SE of −0.1 ± 0.4 D as manifest refraction (all *p* > 0.05 as compared to 6 months). For the residual SE, 57.7% of eyes had a residual error of ±0.25 D, and 80% had ±0.50 D (Figure 2C).

### 3.2. Safety

**Figure 3:**
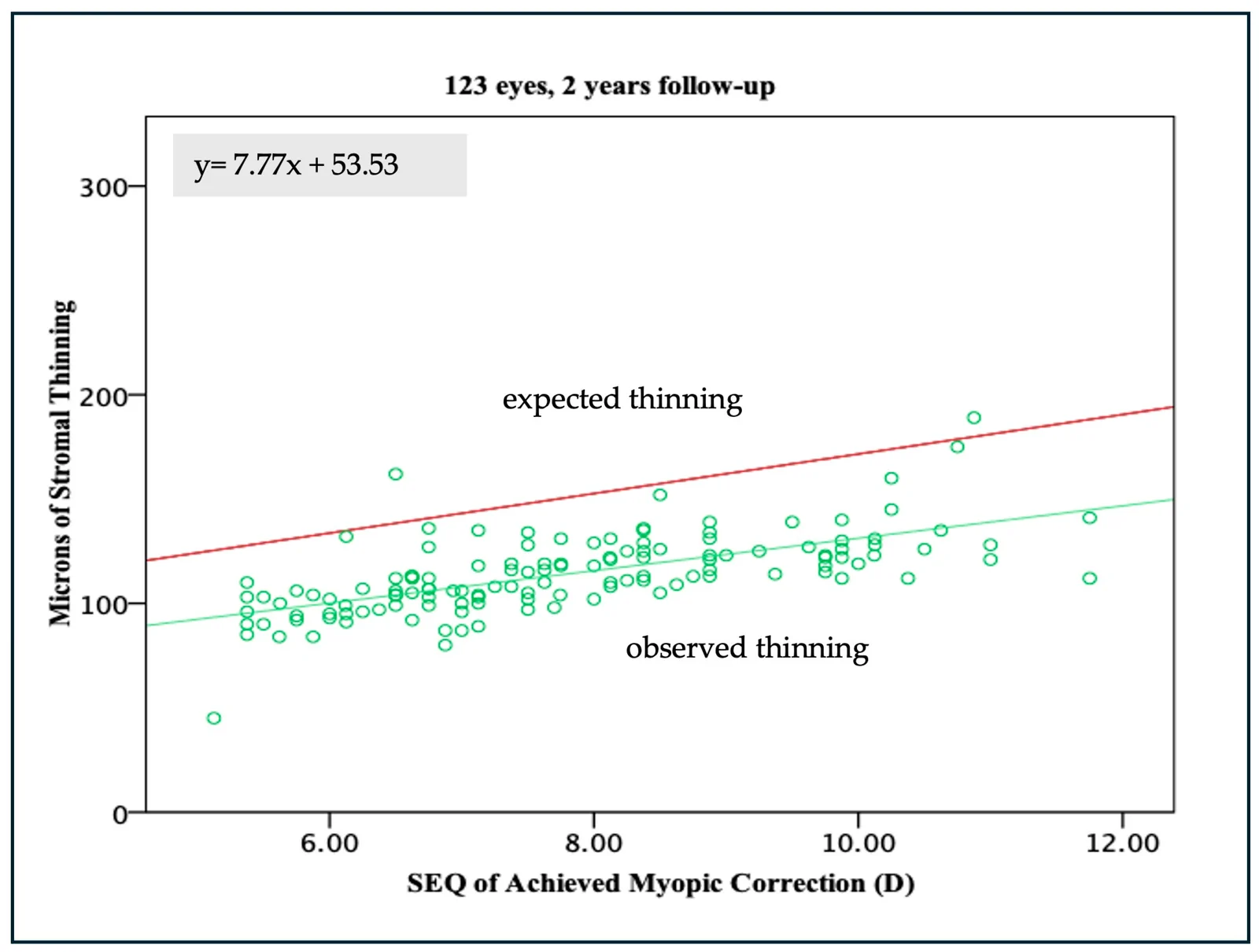
Correlation between stromal thinning and SE diopters of achieved myopic correction. The red line represents the predicted thinning calculated by the Ziemer Z8 Software for Corneal Lenticule Extraction for Advanced Refractive correction (CLEAR). The green line and dots represent the observed stromal thinning.

All the procedures were completed with no complications, and no loss of suction was observed. Only 10% of eyes lost one line of CDVA, and no eyes lost two or more lines (Figure 2B). The expected RSB calculated by the Ziemer Z8 software was 242 ± 10 µm. Interestingly, at POD1 the observed RSB was 274 ± 17 µm and significantly higher than expected (*p* < 0.001). The mean difference between expected and observed RSB was 34 ± 10 µm (*p* < 0.001). As compared to POD1, no significant differences were observed for the RSB at 6 months (266.8 ± 16 µm, *p* > 0.05) and 2 years follow-up (265.7 ± 17 µm, *p* > 0.05). At 2 years, the observed RSB was 30.3 ± 8.7 µm thicker than the expected RSB (*p* < 0.001). Indeed, the stromal thinning at 6 months was significantly lower than the value predicted by the software (103.1 ± 18 µm vs. 134.7 ± 25 µm, respectively, *p* < 0.001), and this parameter remained stable at 2 years (100.3 ± 18 µm, *p* > 0.05) (Figure 3). Simple linear regression was used to test if corrected SE diopters significantly predicted stromal thinning. The fitted regression model was: stromal thinning = 53.53 + 7.77 × (corrected diopters of SE), and the overall regression was statistically significant (*p* < 0.001).

### 3.3. Tomography Changes

**Figure 4:**
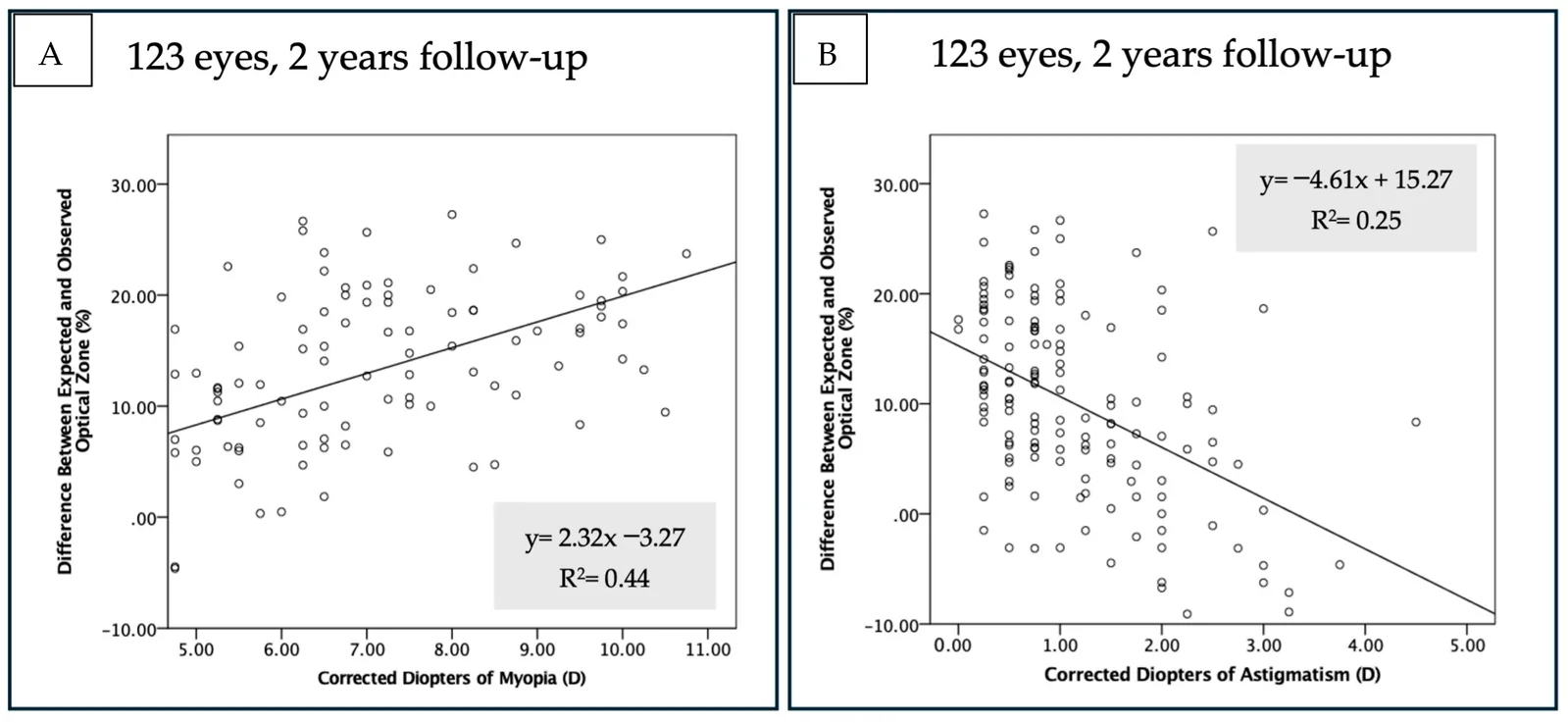
Difference between predicted (POZ) and effective optical zone (EOZ). (**A**): The scatter plot shows that the difference between POZ and EOZ significantly correlated with the degree of myopia corrected. (**B**): The scatter plot shows an inverse correlation between the degree of astigmatism correction and the difference between POZ and EOZ. Patients with high astigmatism (>2 D) can achieve a larger EOZ compared to the POZ.

Corneal parameters and changes were assessed with MS-39 AS-OCT. At POD1, epithelial thickness was significantly higher than pre-operative values (64.1 ± 4 vs. 55.3 ± 4 µm, *p* = 0.013), and it did not change at 6 months and 2 years follow-up (64.1 ± 5 µm and 64.5 ± 5 µm, respectively, *p* > 0.05). As expected, the mean anterior corneal curvature was significantly flatter at 2 years as compared to the preoperative assessment (36.2 ± 2 vs. 44.7 ± 0.3 D, *p* < 0.001). Simple linear regression was used to test if corrected SE diopters significantly predicted changes in the anterior corneal curvature. The fitted regression model was: anterior corneal curvature = 31.51 + 0.743 × (corrected diopters of SE), and the overall regression was statistically significant (*p* = 0.025). The EOZ calculated at 2 years follow-up was 11% smaller than the predicted optical zone (POZ) by the Ziemer Z8 software (5.4 ± 0.5 vs. 6.1 ± 0.3 mm, *p* < 0.001). Moreover, the difference between POZ and EOZ significantly correlated with the degree of myopia corrected (r = 0.6, *p* < 0.001). Interestingly, the difference between POZ and EOZ inversely correlated with astigmatism correction (r = −0.5, *p* < 0.001), with some patients having a larger OZ than that predicted by the software for astigmatisms higher than −2 D (Figure 4).

## 4. Discussion

In this paper, we demonstrated that CLEAR surgery is a safe and effective procedure in patients with high myopia. CLEAR has recently been introduced as corneal lenticule procedure to correct myopia and astigmatism, demonstrating promising results [9,12,13,14]. Among the other KLEx procedures, the femtosecond laser Ziemer Z has the peculiarity of using low-energy pulses to minimize corneal inflammation and stromal gas formation [16,17,18]. In our first paper [12], we showed good refractive results of CLEAR surgery in patients with myopia and astigmatism, although visual recovery was slower than what observed with femto-LASIK. However, that was our first experience with KLEx surgery, and since then we have optimized our surgical technique and nomogram. Furthermore, in our first paper we included patients with amblyopic eyes, a factor that might account for the slow visual recovery after surgery. In this work, our clinical results show that CLEAR allows us to correct high myopic defects with a faster visual recovery, good optical zones, and low impact on corneal thickness.

As previously demonstrated [5,8], the main advantage of the KLEx technique over femto-LASIK lies in its ability to correct refractive errors without the need to create a corneal flap. The flap, in fact, not only carries a risk of displacement following trauma [11,19], but also has the disadvantage of altering corneal biomechanics [20]. Indeed, as shown in previous studies, the vertical incisions at the flap edge appear to weaken the corneal stroma more than the horizontal incisions used in KLEx [21]. Furthermore, because the cohesive strength of the collagen lamellae is greater in the anterior stroma, this region is more affected in femto-LASIK than in KLEx [22]. Finally, since the incision is performed at a deeper stromal level than in femto-LASIK, KLEx has been shown to reduce the incidence of iatrogenic dry eye and to allow faster regeneration of corneal nerves [5].

Many previous studies have assessed the efficacy of high myopia correction with SMILE [23,24,25,26,27]. Reinstein et al. showed that UDVA was 20/25 or better in 82% of the eyes at 12 months with a mean SE of −0.08 ± 0.34 D [27]. In our published study on CLEAR [12], we included patients with up to −10 D of myopia, and we observed a postoperative UDVA of 20/25 or better at 6 months in 70% of treated subjects. Bteich et al. assessed CLEAR results for myopia correction at 1 year, and they found that more than 95% of patients had a visual acuity equal or greater than 20/20 [14]. However, to our knowledge, no published works have assessed CLEAR results for high myopia correction at 2 years follow-up. In the present paper, we observed a good UCVA at POD 1, which confirms the low impact of Ziemer Z8 femtosecond laser on corneal inflammation postoperatively. Furthermore, more than 95% of subjects included in the study were able to see 20/25 or better at 6 months, and the value remained stable at 2 years of follow-up. Indeed, our study shows for the first time that CLEAR surgery is safe and effective in correcting high myopia, with a very good long-term stability and a low incidence of myopic regression. The long-term refractive stability observed in this study is particularly relevant in high myopia. Correction of high myopia has historically been associated with reduced predictability and a greater tendency toward residual myopia or postoperative regression after corneal refractive surgery. In KLEx, high corrections require a thicker lenticule and produce greater changes in anterior corneal curvature, while postoperative epithelial and stromal remodeling may further influence the final refractive result [20]. Previous SMILE studies have nevertheless shown that satisfactory stability can be maintained in highly myopic eyes, including during long-term follow-up [23,24,25,26,27,28]. In our cohort, the mean residual SE was −0.12 D at 6 months and −0.10 D at 24 months, with no significant change between these visits. Moreover, the attempted-versus-achieved relationship remained strong at 2 years, and 80% of eyes were within ±0.50 D of emmetropia. Taken together, these results indicate that the refractive correction achieved at 6 months was maintained through the 2-year follow-up, despite the high degree of myopia treated. Although direct comparisons cannot be made in the absence of a control group, the stability observed in this study appears consistent with the long-term experience reported for SMILE in high myopia [25,28].

Another very interesting finding from this study concerns the residual stromal thickness. In another paper published by our group [9], the corneal thinning resulting from CLEAR lenticule extraction was less than that predicted by the software. In the present paper, although the high refractive defect was treated, the observed residual stroma was on average 30 µm thicker than the predicted value. These results correlate with SMILE studies, and confirm that CLEAR surgery can be safely performed to correct high myopia [28,29]. Many studies have demonstrated that KLEx induces less biomechanical changes in cornea as compared to femto-LASIK [30,31,32], but evidence remains limited. Indeed, Raevadal et al. [33] showed in a systematic review that only non-randomized studies have shown a difference in terms of corneal hysteresis and corneal resistance factor (CRF) between cap-based and flap-based procedures, whereas randomized controlled studies have not found a statistically significant difference. Hashemi et al. [34] found that SMILE preserves a greater part of the anterior cornea, but it also leads to a reduced residual stromal bed and a decreased corneal stiffness. Thus, it is no wonder that PRK has the lowest decrease in corneal hysteresis although more anterior stroma is consumed, since the preserved RSB is higher than the other two procedures [34]. Thus, in this scenario it is evident that preservation of the RSB is crucial to reduce the impact of the refractive procedures on corneal stiffness and hysteresis. In our study, CLEAR showed excellent results in terms of preservation of the RSB, and these data correlate with the findings reported in our previous paper [9]. Moreover, we also found a significant difference between observed and predicted RSB, suggesting that the actual stromal tissue removal may be lower than that predicted by the current software model. This finding may be relevant when planning CLEAR in highly myopic eyes, although established RSB safety limits should continue to guide patients’ selection.

In this study, we demonstrated that CLEAR surgery leads to a larger optical zone as compared to femto-LASIK [15]. Additionally, patients with higher astigmatism achieve a larger EOZ than subjects with low astigmatism, as previously demonstrated by other studies [35,36,37]. It seems that high astigmatism may cause smaller changes in the corneal peripheral contours and volume because the anterior collagen fibers are preserved [38], reducing the peripheral stromal matrix relaxation and producing a larger EOZ. A reduction in the EOZ relative to the programmed optical zone is commonly observed after myopic corneal refractive surgery; this difference tends to increase with the magnitude of myopic correction [15,35,36,37]. This is relevant because the optical zone represents not only a geometric treatment parameter but also a determinant of postoperative optical quality. Previous studies of SMILE, LASIK and PRK have associated smaller effective or functional optical zones with greater induction of spherical aberration and other higher-order aberrations [35,36]. In high myopia, the balance between preserving an adequate optical zone and limiting stromal tissue consumption is particularly important, because increasing the programmed optical zone also increases the amount of tissue required for correction. In the present study, the mean EOZ at 2 years was 5.4 mm compared with a mean programmed optical zone of 6.1 mm, and the discrepancy increased with the amount of myopia corrected. This finding supports the need to consider the expected postoperative EOZ, rather than the programmed diameter alone, during preoperative planning in highly myopic eyes. At the same time, the inverse association between astigmatic correction and the POZ-EOZ difference is of particular interest. Eyes receiving larger cylindrical corrections tended to maintain a relatively larger EOZ, and in some eyes with astigmatism greater than 2 D the measured EOZ exceeded the programmed value. Similar relationships between astigmatism and functional optical-zone geometry have been reported after SMILE [35,36,37]. The preservation of a larger EOZ in these eyes may be related to the non-symmetric lenticule geometry required for cylindrical correction and to different patterns of peripheral stromal relaxation [38].

From a clinical perspective, our results suggest that CLEAR may represent a valuable option for the correction of high myopia. In these patients, preservation of corneal tissue is particularly important, since the amount of stromal tissue required for the correction may limit the possibility of performing corneal refractive surgery. In our study, the observed RSB was approximately 30 µm thicker than that predicted by the software at 2 years, despite the high degree of myopia treated. This finding, together with the good refractive stability observed during follow-up, suggests that CLEAR may be particularly useful when stromal preservation is an important factor in surgical planning. As a KLEx procedure, CLEAR also avoids the creation of a corneal flap, with the previously described advantages in terms of preservation of the anterior stromal architecture and corneal innervation compared with femto-LASIK [5,20,21,22].

Some characteristics of the Ziemer Z8 platform may offer additional advantages in clinical practice. CLEAR uses corneal applanation associated with scleral suction and double vacuum control, providing stable fixation during the procedure. A low incidence of suction loss has been previously reported with this system, and no suction loss occurred in our series [39]. The large corneoscleral patient interface also allows treatment over a relatively large corneal area, which may be useful when larger treatment zones are required [12,14]. This aspect may be particularly relevant in patients with high myopia and astigmatism, where maintaining an adequate effective optical zone may be challenging. In our study, although the difference between predicted and effective optical zone increased with the degree of myopia corrected, higher astigmatic corrections were associated with a relatively larger EOZ.

This study has several strengths, including a relatively large and homogeneous cohort of eyes with high myopia, a long follow-up period of up to 24 months, and a comparatively low rate of loss to follow-up. However, some limitations should be acknowledged. First, this was a retrospective, single-center study without a randomized comparison group. Second, all procedures were performed by experienced surgeons at a single referral center using a specific nomogram, which may limit the generalizability of our findings to centers with less surgical experience or different treatment protocols. Third, patient-reported outcomes, and in particular satisfaction, were not formally assessed with validated questionnaires. Finally, 14 eyes (10.2%) were lost to follow-up by 2 years, but we consider it a relatively low attrition rate for a retrospective study with a long follow-up.

Thus, preservation of the RSB, the absence of a corneal flap, stable suction and the possibility of treating a relatively large corneal area may make CLEAR an interesting alternative for selected patients with high myopia. Moreover, our study shows excellent refractive stability over a 2-year follow-up period.

## 5. Conclusions

In conclusion, CLEAR surgery is a safe and effective procedure for the correction of high myopia, demonstrating good refractive stability over a 2-year follow-up. In eyes with high astigmatism, CLEAR achieves a larger optical zone, potentially reducing spherical aberrations and improving visual quality. Further prospective randomized studies are warranted to confirm these findings, and to compare CLEAR with femto-LASIK, PRK, SMILE or other KLEx procedures. 

## Data Availability

The data are available on request from the corresponding author.
